# Kaiso depletion attenuates the growth and survival of triple negative breast cancer cells

**DOI:** 10.1038/cddis.2017.92

**Published:** 2017-03-23

**Authors:** Blessing I Bassey-Archibong, Lyndsay G A Rayner, Shawn M Hercules, Craig W Aarts, Anna Dvorkin-Gheva, Jonathan L Bramson, John A Hassell, Juliet M Daniel

**Affiliations:** 1 Department of Biology, McMaster University, Hamilton, Ontario L8S 4K1, Canada; 2 Department of Pathology and Molecular Medicine, McMaster University, Hamilton, Ontario L8S 4K1, Canada; 3 Department of Biochemistry and Biomedical Sciences, McMaster University, Hamilton, Ontario L8S 4K1, Canada

## Abstract

Triple negative breast cancers (TNBC) are highly aggressive and lack specific targeted therapies. Recent studies have reported high expression of the transcription factor Kaiso in triple negative tumors, and this correlates with their increased aggressiveness. However, little is known about the clinical relevance of Kaiso in the growth and survival of TNBCs. Herein, we report that Kaiso depletion attenuates TNBC cell proliferation, and delays tumor onset in mice xenografted with the aggressive MDA-231 breast tumor cells. We further demonstrate that Kaiso depletion attenuates the survival of TNBC cells and increases their propensity for apoptotic-mediated cell death. Notably, Kaiso depletion downregulates BRCA1 expression in TNBC cells expressing mutant-p53 and we found that high Kaiso and BRCA1 expression correlates with a poor overall survival in breast cancer patients. Collectively, our findings reveal a role for Kaiso in the proliferation and survival of TNBC cells, and suggest a relevant role for Kaiso in the prognosis and treatment of TNBCs.

Triple negative breast cancers (TNBC) represent a heterogeneous subtype of breast tumors that generally lack expression of estrogen **r**eceptor (ER), progesterone receptor (PR) and the human epidermal growth factor receptor 2.^1^ TNBCs are highly proliferative and have a high rate of recurrence compared to other breast cancer (BCa) subtypes.^2^ Currently, there are no specific targeted therapies for the management of TNBC, hence treatment is limited to radio- and chemotherapy. Although TNBCs initially respond to chemotherapy, many patients relapse and this contributes to a shortened overall survival for affected patients.^3^

Various proteins have been implicated in the survival and chemo-resistant nature of TNBC. Two of the most understood are the tumor suppressors BRCA1 and p53.^4, 5, 6^ BRCA1 is mutated in ~45% of familial BCa^7^ and a high proportion of sporadic BCa, especially of the TNBC subtype.^8, 9^ However, some TNBCs retain the expression of wild-type (wt) BRCA1 (which plays a role in DNA repair) and this has been associated with their resistance to chemotherapeutic drugs such as Cisplatin.^10^ Similarly, p53 is mutated in ~30% of BCa^11^ with a higher frequency observed in TNBCs, reviewed in Walerych *et al.*^12^ The inability of mutant p53 to bind and activate the expression of canonical p53 target genes such as the pro-apoptotic genes Noxa, Bax and Puma is believed to contribute to the chemo-resistance and survival of BCa.^13, 14, 15, 16, 17, 18^

Several recent studies have implicated increased nuclear expression of the transcription factor Kaiso in the aggressiveness of certain tumors including basal/triple negative tumors.^19, 20^ Kaiso is a POZ-ZF transcription factor that was first identified as a binding partner of the E-cadherin catenin cofactor p120-catenin (p120^ctn^).^21^ In the absence or downregulation of E-cadherin, p120^ctn^ is able to translocate to the nucleus^22, 23^ where it can bind and inhibit Kaiso's transcriptional activities.^23, 24, 25^ Although high Kaiso expression is associated with TNBC aggressiveness,^19, 26^ Kaiso's specific role in the growth and survival of TNBCs remains unknown. Interestingly, roles for Kaiso in cell growth (proliferation) and survival (decreased apoptosis) have been demonstrated in several cell types. For instance, Kaiso depletion results in decreased Cyclin D1, reduced proliferation and increased apoptosis of cervical cancer (HeLa) cells, but decreased apoptosis of human embryonic kidney (HEK293) cells.^27^ Similarly, loss of Kaiso decreased prostate tumor cell proliferation^20^ and delayed the onset of intestinal polyp formation in *Apc*^Min+^ mice.^28^ Finally, loss of Kaiso-mediated transcriptional repression is associated with increased anchorage-independent cell growth of mouse lobular BCa cells.^23^ Collectively, these studies suggest context-dependent roles for Kaiso in cell proliferation and apoptosis.

Herein, we report that Kaiso depletion attenuates the proliferative ability of TNBC cells, reduces the anchorage-independent growth of MDA-231 cells and delays the tumor onset of MDA-231 xenografts. We also show that Kaiso depletion increases the apoptosis of TNBC cells. More importantly, we report for the first time that silencing Kaiso results in the downregulation of BRCA1 in mutant-p53-expressing TNBC cells. Together, our findings suggest that high Kaiso expression promotes the growth and survival of TNBCs and raise the possibility that Kaiso may be a useful biomarker for the prognosis and treatment of a subset of TNBCs.

## Results

### Kaiso depletion inhibits TNBC cell proliferation

**Figure 1:**
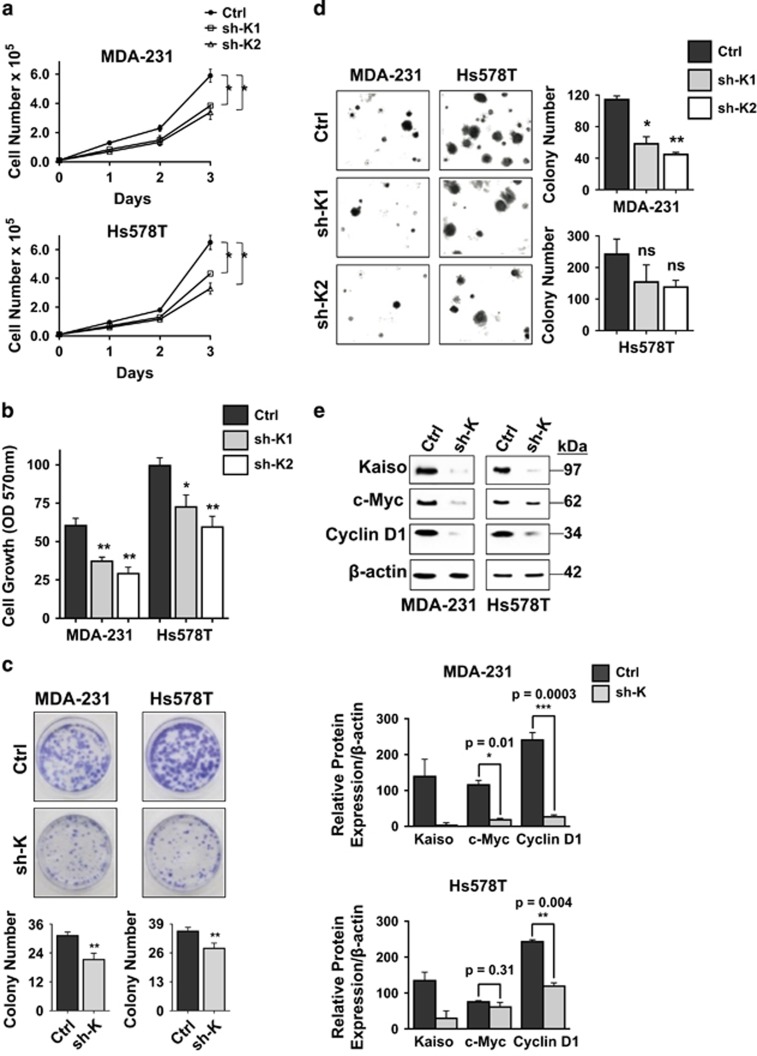
Kaiso depletion attenuates the proliferation of TNBC cells. Growth curve (**a**), MTT assays (**b**) and colony formation assays (**c**) were conducted on Ctrl and Kaiso-depleted (sh-K1 and sh-K2, hereafter referred to as sh-K) cells, and reveal that Kaiso depletion inhibits proliferation of MDA-231 and Hs578T cells. (**d**) Soft agar assays were also conducted on Ctrl, sh-K1 and sh-K2 MDA-231 and Hs578T cells and revealed that Kaiso-depletion diminished anchorage-independent growth of MDA-231 but not Hs578T cells. (**e**) Kaiso depletion resulted in decreased c-Myc and cyclin D1 expression in MDA-231 and Hs578T cells as detected by IB analysis and densitometry analysis. The reduction in c-Myc levels in response to Kaiso depletion was more significant in MDA-231 compared to Hs578T cells. GraphPad Prism software was used to generate graphs and for all statistical calculations. Data shown are representative of three independent experiments. *P<0.05, **P<0.01 and ***P<0.001

Recently, we reported that Kaiso is highly expressed in triple negative tumors and correlates with the metastatic propensity of TNBC cells.^19, 26^ To ascertain whether Kaiso is also involved in TNBC cell growth and survival, we performed cell viability assays (direct cell counts, MTT and colony formation assays) on control (Ctrl) and Kaiso-depleted (sh-K1 and sh-K2, hereafter referred to as sh-K) TNBC cell lines (MDA-231 and Hs578T), see Bassey-Archibong *et al.*^26^ Notably, silencing Kaiso significantly inhibited the proliferation of MDA-231 and Hs578T cells in all assays performed (Figures 1a–c). We also conducted soft agar assays on Ctrl and Kaiso-depleted MDA-231 and Hs578T cells, and found that Kaiso-depletion mitigates the anchorage independence of MDA-231 but not Hs578T cells (Figure 1d). As previous studies had implicated Kaiso in the regulation of the pro-proliferation genes *c-myc* and *cyclin D1*,^24, 29, 30^ we examined the expression of these proteins in Kaiso-depleted TNBC cells. We found that Kaiso-depleted MDA-231 and Hs578T cells expressed less c-Myc and Cyclin D1 than control cells (Figure 1e and Supplementary Figure 1A), which further supported a role for Kaiso in cell proliferation.

### Kaiso depletion results in delayed tumor onset of MDA-231 xenografts

**Figure 2:**
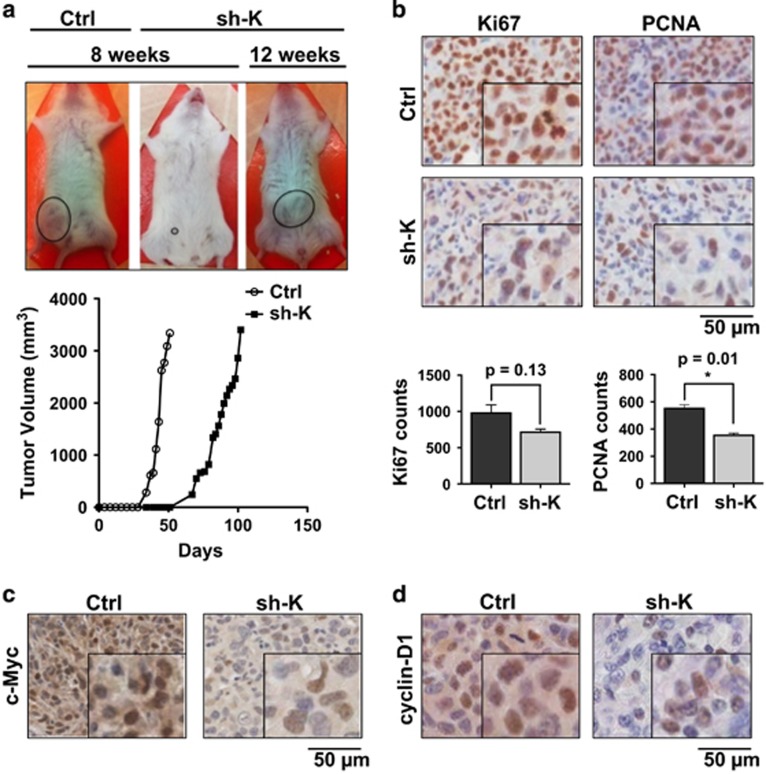
Kaiso-depleted MDA-231 cells exhibit delayed tumor onset in mouse xenografts. (**a**) Kaiso-depleted MDA-231 xenografts (sh-K) are delayed ~3 weeks in tumor onset and development compared to control (Ctrl) MDA-231 xenografted tumors as seen by time-course analysis of the tumor volume of Ctrl and sh-K MDA-231 xenografted cells. (**b**) IHC-stained images of MDA-231 xenograft tissues with Ki-67 and PCNA antibodies show a marked decrease in proliferating cells in MDA-231 Kaiso-depleted tumor tissues as indicated by the reduced expression of the proliferation markers Ki-67 and PCNA. (**c** and **d**) IHC-stained images of MDA-231 xenograft tissues with c-Myc and Cyclin D1 antibodies show that Kaiso-depletion results in reduced numbers of c-Myc and cyclin-D1 stained cells and reduced staining intensity. Representative images shown from 3 or more independent experiments

To ascertain whether the *in vitro* effect of Kaiso depletion on TNBC cell proliferation would be sustained *in vivo*, we performed mouse xenograft studies with the well-characterized TNBC cell line–MDA-231. Equal numbers (4.5 × 10^6^) of Ctrl and Kaiso-depleted (sh-K) MDA-231 cells were injected into the mammary fat pad of immunocompromised mice (*n*=5 for each experimental condition) and allowed to form tumors as previously described.^26^ Interestingly, we observed a significant delay (~8 weeks) in tumor formation in the Kaiso-depleted xenografts compared to controls which took ~5 weeks to form visible tumors (Figure 2a). Furthermore, upon tumor formation, the Kaiso-depleted tumors took ~4 weeks to reach the endpoint size of 3300 mm^3^ compared to the control tumors which took ~3 weeks to reach 3300 mm^3^ (Figure 2a). To determine if the delayed tumor onset observed in the Kaiso-depleted xenografts was due to Kaiso-depletion effects on proliferation, size-matched (~3300 mm^3^) Ctrl and sh-K MDA-231 tumor tissues were harvested and examined for the expression of the well-established proliferation markers (Ki-67 and PCNA). Immunohistochemical (IHC) analyses revealed less proliferating cells in the Kaiso-depleted tumor tissues compared to control tissues (Figure 2b and see Bassey-Archibong *et al.*^26^ for IHC analysis of Kaiso expression in the Ctrl and sh-K tumor tissues). This suggests that the delayed tumorigenesis of the Kaiso-depleted MDA-231 cells may be due to their reduced proliferative capacity. However, the delayed tumor onset may also be due to the reduced colonization of Kaiso-depleted MDA-231 cells, since these cells displayed decreased anchorage-independence *in vitro* (Figure 1d). Nonetheless, consistent with our *in vitro* proliferation studies, IHC analysis revealed reduced c-Myc and Cyclin D1 expression in Kaiso-depleted MDA-231 tumors compared to control MDA-231 tumor tissues (Figures 2c and d). Collectively, these findings further support a role for Kaiso in TNBC cell proliferation.

### Kaiso depletion induces apoptosis in TNBC cells

**Figure 3:**
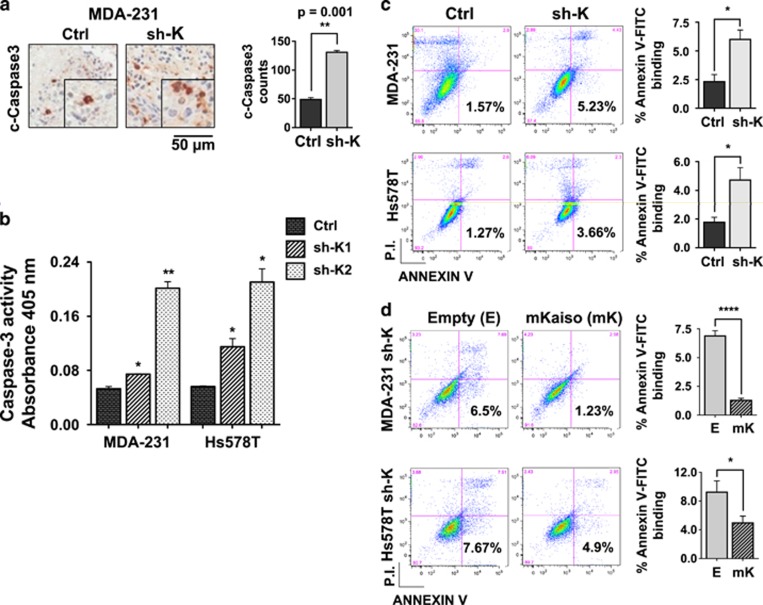
Kaiso depletion results in increased apoptosis of TNBC cells. (**a**) IHC-stained images of control (Ctrl) and Kaiso-depleted (sh-K) MDA-231 tumor tissues with cleaved (c)-Caspase 3 antibody show that Kaiso-depletion results in increased c-Caspase 3 expression in MDA-231 tumor tissues. (**b**) Caspase 3 assay conducted on Ctrl, sh-K1 and sh-K2 MDA-231 and Hs578T cells show that Kaiso depletion resulted in increased Caspase 3 activity in MDA-231 and Hs578T sh-K1 and sh-K2 cells compared to their control counterparts. (**c**) Kaiso-depleted TNBC cells (sh-K MDA-231 & Hs578T) expressing mut-p53 exhibit increased apoptosis as revealed by Annexin V-FITC staining. (**d**) Expression of a sh-resistant Kaiso cDNA in Kaiso-depleted MDA-231 and Hs578T cells mitigates the apoptosis induced by Kaiso depletion as assessed by Annexin V-FITC staining. Data shown are representative of three independent experiments. *P<0.05, **P<0.01, ****P<0.0001

As the delay in MDA-231 tumor onset could also have been due to increased apoptosis in Kaiso-depleted cells, we investigated the effect of Kaiso depletion on the expression of the apoptotic/cell-death marker–cleaved Caspase 3 (c-Caspase 3) in MDA-231 tumor tissues. Remarkably, we observed an increased number of c-Caspase 3 stained cells in Kaiso-depleted MDA-231 tumors compared to control MDA-231 tumors (Figure 3a). Quantification of the Caspase 3 activity of control and Kaiso-depleted (sh-K1 & sh-K2) MDA-231 cells *in vitro,* using the Caspase 3 colorimetric assay, also revealed increased Caspase 3 activity in the Kaiso-depleted (sh-K1 & sh-K2) MDA-231 cells compared to control cells (Figure 3b). Similar results were also observed in Kaiso-depleted (sh-K1 & sh-K2) Hs578T cells compared to their control counterparts (Figure 3b). Further verification of Kaiso depletion effects on apoptosis with the Annexin V-fluorescein isothiocyanate (FITC) staining assay also confirmed that Kaiso depletion resulted in increased apoptosis of MDA-231 and Hs578T cells as evidenced by the elevated number of Annexin V-FITC stained cells in Kaiso-depleted (sh-K) cells compared to controls (Figure 3c). Similar results were also obtained in an additional TNBC cell line–MDA-157 (Supplementary Figure 2A). To determine if the increased apoptosis in the TNBC cells was specific to Kaiso depletion, we expressed a sh-resistant murine Kaiso cDNA (mKaiso) in the MDA-231 and Hs578T sh-K cells, and subjected these cells to Annexin V-FITC staining. As observed in Figure 3d, Kaiso re-expression rescued the apoptotic phenotype observed in the Kaiso-depleted (sh-K) MDA-231 and Hs578T cells, as seen by the reduced number of Annexin V-FITC stained cells in the MDA-231 and Hs578T sh-K (mK) cells compared to Kaiso-depleted MDA-231 and Hs578T cells transfected with an empty (E) vector. Together these findings suggest that silencing Kaiso enhances the apoptosis of TNBC cells.

Our observation that Kaiso depletion caused increased apoptosis in TNBC cells was intriguing but contradictory to recent findings in other cell types (MEF and HEK293) where Kaiso was implicated as a pro-apoptotic protein, and promoter of p53-mediated apoptosis.^31^ Since the TNBC cells utilized in this study (MDA-231, Hs578T, MDA-157) contain a mutant (mut)-p53 gene^32, 33, 34^ compared to MEF and HEK293 cells that express wt-p53,^31^ we postulated that Kaiso's role in apoptosis was contingent on the status of p53 rather than cell type per se. To test this hypothesis, we performed Annexin V-FITC staining of MCF-7 cells transiently overexpressing Kaiso. The MCF-7 BCa cell line was selected for these studies as it expresses low levels of Kaiso and wt-p53. As seen in Supplementary Figure 2B, transient overexpression of Kaiso in MCF-7 cells enhanced the apoptosis/death of these cells, as evidenced by more Annexin V-FITC stained cells in the Kaiso-overexpressing (mKaiso) MCF-7 cells compared to their parental (empty) counterparts, consistent with the findings of Koh *et al.*^31^

### Pro-apoptotic proteins are up-regulated in Kaiso-depleted TNBC cells

**Figure 4:**
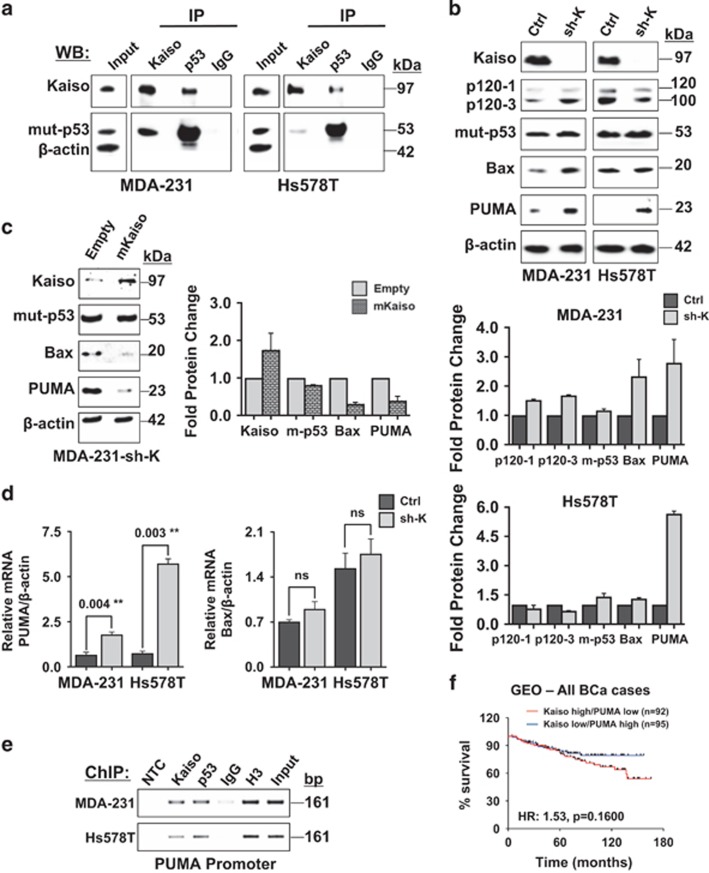
Kaiso depletion increases expression of pro-apoptotic proteins in TNBC cells lacking wt p53. (**a**) Kaiso co-precipitates with mutant p53 in MDA-231 and Hs578T cell lysates. Parental MDA-231 and Hs578T cells were subjected to immunoprecipitation with anti-Kaiso, anti-p53 and anti-IgG antibodies, and immunoblotted with the indicated antibodies. Kaiso-depleted MDA-231 and Hs578T cells express increased amounts of Bax and PUMA protein compared to control cells (**b**), that is decreased upon expression of a sh-resistant Kaiso cDNA in the MDA-231 and Hs578T sh-K cells (**c**). Graphical representation of the quantitated protein values is shown. (**d**) Kaiso-depleted MDA-231 and Hs578T cells (that express mut-p53) exhibit a statistically significant increase in PUMA mRNA levels as measured by qRT-PCR. Although Bax mRNA levels were also slightly increased, it was not significant. (**e**) ChIP-PCR analysis of MDA-231 and Hs578T chromatin revealed that Kaiso and mut-p53 associate endogenously with the PUMA promoter in TNBC cells. (**f**) Transcript profiles of patients from the GEO (GSE20685, GSE21653, GSE16446, GSE19615 and GSE9195) BCa data sets were pooled and segregated into Kaiso high/PUMA low, and Kaiso low/PUMA high groups. Kaplan–Meier survival curves revealed a correlation between high Kaiso and low PUMA expression with poor overall survival in all BCa cases. However, it was not statistically significant. Data representative of three independent experiments. **P<0.01 and NS, not significant. For Kaplan–Meier survival curves, log-rank test was performed to determine statistical significance. *P*<0.05 is considered statistically significant

As Kaiso expression promotes survival in TNBC cells expressing mut-p53 (Figure 3c), we hypothesized that the pro-survival role of Kaiso in TNBC cells is due to its interaction with mut-p53. To test this hypothesis, we performed co-immunoprecipitation experiments and found that Kaiso associated with mut-p53 in MDA-231 and Hs578T cells, although a stronger interaction was observed between Kaiso and mut-p53 in MDA-231 compared to Hs578T cells (Figure 4a). Wt-p53-expressing MCF-7 cells were also examined as a positive control to confirm Kaiso's interaction with wt-p53 (Supplementary Figure 3A) as previously reported.^31^

To gain more mechanistic insight into Kaiso's role in TNBC cell survival and apoptosis, we assessed the effect of Kaiso depletion on the expression of the pro-apoptotic proteins PUMA and Bax. As Kaiso augments the expression of PUMA and Bax in wt-p53-expressing cells^31^ and (Supplementary Figure 3B), we postulated that in mut-p53-expressing TNBC cells, Kaiso would inhibit PUMA and Bax expression. Indeed, low levels of PUMA were detected in control MDA-231 and Hs578T cells that express high levels of Kaiso and mut-p53 (Figure 4b). PUMA was also detected at low levels in the high Kaiso and mut-p53 MDA-157 cells (Supplementary Figure 4). Similarly, low Bax levels were detected in control MDA-231 and MDA-157 cells (Figure 4b and Supplementary Figure 4) but not control Hs578T cells (Figure 4b). Remarkably, Kaiso depletion resulted in a striking upregulation of PUMA in all three cell lines (MDA-231, Hs578T and MDA-157 cells; Figure 4b and Supplementary Figure 4). While Bax expression was increased ~2-fold in MDA-231 and MDA-157 cells, there was only a slight increase in Bax expression in Hs578T cells (Figure 4b and Supplementary Figure 4), which may be due to the fact that Bax was expressed at higher levels in parental Hs578T cells compared to parental MDA-231 and MDA-157 cells (Figure 4b and Supplementary Figure 4). This suggests that Kaiso may not exhibit a repressive function on Bax expression in parental (Ctrl) Hs578T cells, probably due to the increased expression of p120^ctn^ observed in these cells (Figure 4b), which co-localized with Kaiso in the nucleus of some but not all Ctrl Hs578T cells (Supplementary Figure 5). Notably, there was no change in mut-p53 levels upon Kaiso depletion. The specificity of Kaiso depletion effects on Bax and PUMA protein levels was confirmed by the expression of a sh-resistant Kaiso (mKaiso) cDNA in the MDA-231 sh-K cells; this resulted in reduced Bax and PUMA protein expression in the MDA-231-sh-K (mKaiso) cells (Figure 4c).

Additional analyses using qRT-PCR revealed significantly increased PUMA transcripts but no significant changes in Bax transcript levels in Kaiso-depleted MDA-231 and Hs578T cells compared to controls (Figure 4d). Consistent with this observation, chromatin immunoprecipitation (ChIP)-PCR experiments showed an enrichment of Kaiso at a minimal PUMA promoter region rich in Kaiso binding sequences (KBS) but not at a minimal Bax promoter region containing a core KBS (see schematic, Supplementary Figure 6A) in MDA-231 and Hs578T cells (Figure 4e, and data not shown). Similar results were also obtained with chromatin isolated from MDA-157 cells (Supplementary Figure 6B). Interestingly, an enrichment of mut-p53 was also observed at the minimal PUMA promoter region rich in KBS but not at the minimal Bax promoter region containing a core KBS (Figure 4e, and data not shown). Nonetheless, Kaiso's interaction with the PUMA promoter was independent of p53 as evidenced by its association with the PUMA promoter in MDA-157 cells, which lack detectable p53 protein expression (Supplementary Figure 4). Collectively, these data imply that Kaiso may directly or indirectly inhibit Bax and PUMA expression in TNBC cells that lack wt-p53.

### High Kaiso and low PUMA expression does not correlate with poor survival in BCa patients

Considering the consistent effect of Kaiso depletion on PUMA expression in all TNBC cell lines (MDA-231, Hs578T and MDA-157) studied, we explored whether the inverse correlation of Kaiso and PUMA expression could account for Kaiso's role in the survival of breast tumors. We thus utilized The Cancer Genome Atlas (TCGA) and the Gene Expression Omnibus (GEO) BCa data sets and examined the effect of high Kaiso and low PUMA expression on the overall survival of either TNBC patients specifically (data not shown), or all BCa cases. Kaplan–Meier survival curves revealed that patients bearing tumors with high Kaiso and low PUMA expression exhibited a decreased but non-significant overall survival trend compared to patients with tumors that had a low Kaiso and high PUMA expression (log-rank test, *P*-value=0.16; Figure 4f). This suggests that while Kaiso's effect on PUMA expression does have some effect on BCa survival, the clinical relevance does not appear to be statistically significant. Thus, Kaiso may cooperate with other protein(s) to influence BCa survival.

### Kaiso depletion enhances the sensitivity of TNBC cells to Cisplatin

**Figure 5:**
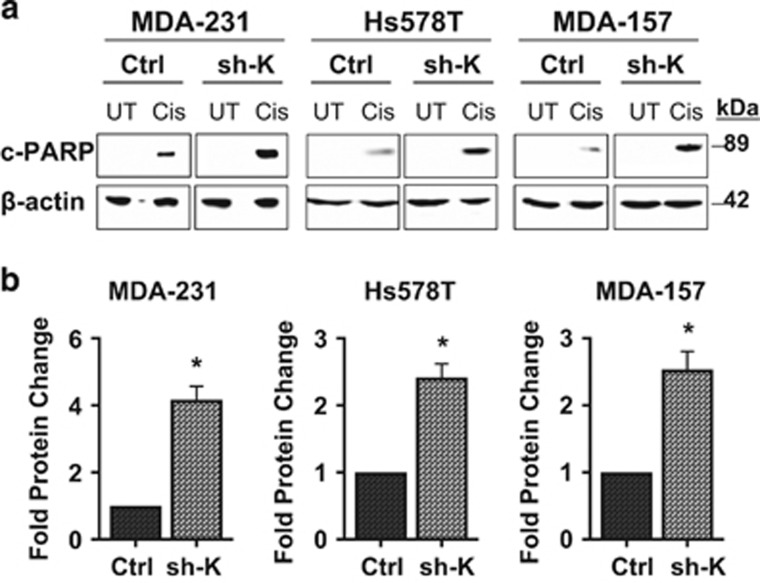
Kaiso depletion enhances the sensitivity of TNBC cells to Cisplatin. (**a**) Kaiso-depletion sensitizes TNBC cells to Cisplatin treatment, as demonstrated by the increased cleaved-PARP expression observed in Kaiso-depleted MDA-231, Hs578T and MDA-157 cells treated with Cisplatin (Cis) for 48 h compared to similarly treated control cells. UT, untreated cells. (**b**) Graphical representation of the fold change in protein expression is shown. All experiments were conducted independently at least three times. Representative images shown. *P<0.05

Most metastatic BCas such as TNBCs are resistant to chemotherapeutic agents,^3^ a phenomenon which may be due to reduced apoptosis and increased DNA repair.^35^ As Kaiso depletion stimulated the apoptosis of TNBC cells, we investigated whether silencing Kaiso would sensitize these cells to chemotherapeutic drugs. Control and Kaiso-depleted TNBC cells were treated with the chemotherapy drug Cisplatin and then subjected to immunoblot (IB) analysis for the expression of the apoptotic marker, cleaved-PARP. Intriguingly, loss of Kaiso enhanced the sensitivity of MDA-231, Hs578T and MDA-157 cells to Cisplatin as evidenced by the increased expression of cleaved-PARP in the treated Kaiso-depleted cells compared to the control-treated cells (Figures 5a and b).

### High Kaiso and BRCA1 expression correlates with poor survival in BCa patients

**Figure 6:**
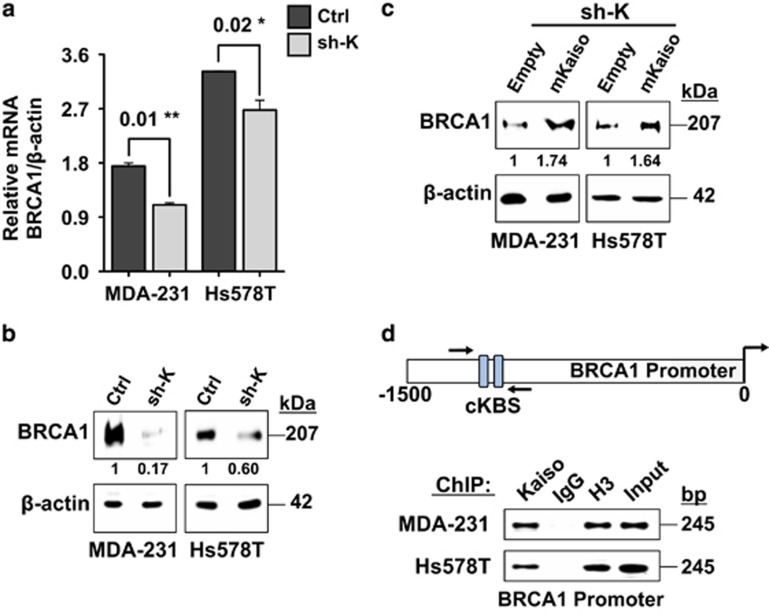
Kaiso depletion attenuates BRCA1 expression in sporadic TNBC cells. (**a**) BRCA1 mRNA expression was significantly reduced in Kaiso-depleted MDA-231 and Hs578T cells compared to controls as measured by qRT-PCR. (**b**) WB analysis with a BRCA1-specific antibody shows decreased BRCA1 protein expression in Kaiso-depleted MDA-231 and Hs578T cells, which is partially rescued by expression of a sh-resistant Kaiso cDNA in the MDA-231 and Hs578T sh-K cells (**c**). (**d**) Schematic illustration of the minimal BRCA1 promoter region showing the location of a core KBS (cKBS) that was amplified by ChIP-PCR. Kaiso was enriched at the BRCA1 promoter region indicated in MDA-231 and Hs578T cells. Data representative of three independent experiments. *P<0.05, **P<0.01

As BRCA1 expression has been linked to the resistance of TNBC cells to Cisplatin,^10, 36^ we examined the effect of Kaiso depletion on BRCA1 expression. We observed that Kaiso depletion led to downregulation of BRCA1 in MDA-231 and Hs578T cells at both the transcript and protein level (Figures 6a and b). This was partially rescued (~1.6-fold increase) by the expression of a sh-resistant Kaiso cDNA (mKaiso) in the Kaiso-depleted MDA-231 and Hs578T cells (Figure 6c). More importantly, we found an enrichment of Kaiso at a minimal BRCA1 promoter region containing several core KBS (Figure 6d), which suggests that BRCA1 may be a Kaiso target gene.

**Figure 7:**
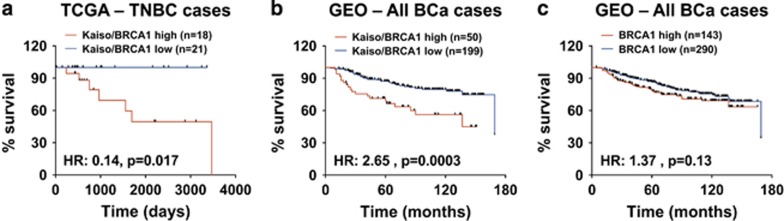
High Kaiso and BRCA1 expression correlates with poor prognosis in BCa patients. Transcript profiles of patients from the TCGA and GEO (GSE20685, GSE21653, GSE16446, GSE19615 and GSE9195) BCa data sets were pooled and segregated into Kaiso/BRCA1 high or Kaiso/BRCA1 low, and BRCA1 high or BRCA1 low groups. Kaplan–Meier survival curves revealed that high Kaiso and BRCA1 expression significantly correlates with poor overall survival in TNBC patients specifically (**a**) or all BCa cases (**b**) whereas increased BRCA1 expression did not correlate with poor overall survival in BCa patients (**c**). Log-rank test was performed to determine statistical significance. *P*<0.05 is considered statistically significant

In light of these findings, we utilized the TCGA and GEO BCa data sets and correlated the expression levels of Kaiso and BRCA1 with BCa survival. Kaplan–Meier survival curves revealed that TNBC patients bearing tumors with high Kaiso and BRCA1 expression, exhibit a significantly worse overall survival compared to TNBC patients with low Kaiso and low BRCA1 expression (log-rank test, *P*=0.017; Figure 7a). A similar trend was also observed in all BCa cases with high Kaiso and high BRCA1 expression (log-rank test, *P*=0.0003) compared to cases with low Kaiso and low BRCA1 expression, and high or low BRCA1 expression alone (log-rank test, *P*=0.13), Figures 7b and c. This finding suggests that Kaiso and BRCA1 function together to promote the survival of BCa cells.

## Discussion

TNBCs remain a clinical challenge due to their highly aggressive nature, lack of specific targeted therapies and resistance to routine chemotherapeutic regimens including anthracyclines and taxanes.^37^ Consequently, there is an urgent need to understand the molecular mechanisms underlying TNBC growth, aggressiveness and chemo-resistance. Herein, we report that depletion of the transcription factor Kaiso attenuates the proliferation of, and increases apoptosis in, the TNBC cell lines MDA-231 and Hs578T. These findings suggest that in addition to Kaiso's potential role in TNBC metastasis,^26^ Kaiso may also be a key regulator of triple negative tumor cell growth and survival.

In the past decade, several independent studies have implicated Kaiso in various cancers; while some studies suggest a pro-oncogenic role for Kaiso,^19, 20, 26, 28, 38, 39, 40, 41, 42^ others associate Kaiso with a tumor suppressive role.^23, 25, 31, 43^ Together, these diverse studies highlight context-dependent roles for Kaiso in human cancer, which might be due to the fact that Kaiso acts as both a transcriptional repressor and an activator.^24, 29, 30, 44, 45^ In addition, as Kaiso also possesses dual-specificity DNA-binding properties,^29, 46, 47^ there may be a large repertoire of tumorigenic target genes that may be differentially regulated by Kaiso. To date, only a few *bona fide* Kaiso target genes—*c-Myc*, *Wnt 11*, *Cyclin D1*, *Siamois*, *Matrilysin* and *Rapysn* have been characterized.^24, 25, 29, 45, 48^ Two of these genes (*c-Myc and Cyclin D1*) are well-established pro-proliferation oncogenes^49, 50^ that were found to be repressed by Kaiso in *Xenopus laevis* embryos and colon cancer cells.^24, 29^ Therefore, it was surprising to find that loss of Kaiso in TNBC cells led to their decreased, rather than increased, expression (Figure 1e). Our findings thus indicate context-dependent roles for Kaiso in the regulation of *c-Myc* and *Cyclin D1* expression, an idea that is supported by a recent study which demonstrates differential regulation of *Cyclin D1* by Kaiso.^27^

Kaiso's role in specific cancers may also be dictated or modulated by its interaction with other transcriptional cofactors or proteins that may be uniquely expressed in these cancers. For example, Kaiso was shown to interact with nuclear p120^ctn^ in mouse invasive lobular BCa cells, which inhibited Kaiso's repression of *Wnt11*, and fostered anoikis resistance in these cells.^23^ In another study, Kaiso was shown to interact with wt-p53, and promote apoptosis through increased p53-mediated expression of the pro-apoptotic *Bax* and *PUMA* genes.^31^ Our findings in this study also support distinct roles of Kaiso that may be based on its interaction with p53, as we found that Kaiso differentially regulates apoptosis in BCa cells that express different forms of p53 (Figure 3c, Supplementary Figures 2A and B). Specifically, Kaiso exhibits an anti-apoptotic role in TNBC (MDA-231, Hs578T and MDA-157) cells that express mut-p53 as its depletion promotes apoptosis in these cells (Figure 3c and Supplementary Figure 2A). As mut-p53 expression is implicated in the survival of MDA-231 and Hs578T cells,^51, 52^ it was interesting to note that loss of Kaiso attenuated the survival of these cells, despite having no significant effects on mut-p53 expression in these cells.

**Figure 8:**
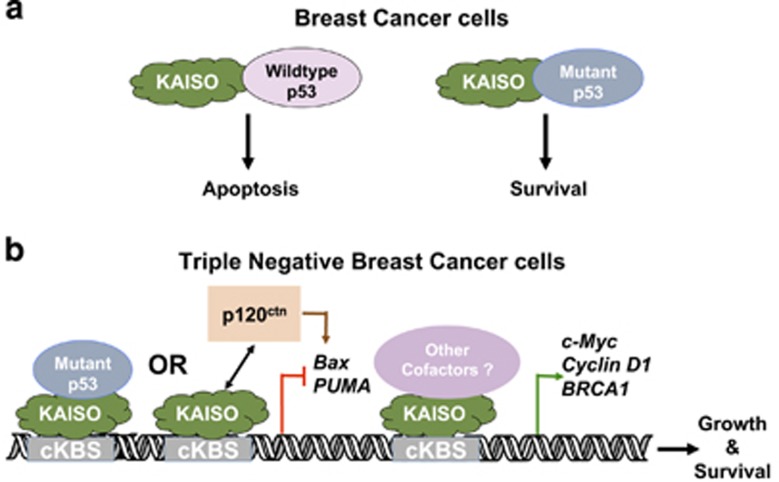
Schematic diagram of proposed model for Kaiso's role in TNBC. (**a**) Kaiso interacts with both wt p53 and mutant p53 in BCa cells and this differential interaction may modulate Kaiso's function in apoptosis. (**b**) In TNBC cells lacking wt p53 (but expressing mutant p53), Kaiso might directly or indirectly inhibit the activation of the pro-apoptotic genes Bax and PUMA, which leads to tumor survival. However, Kaiso's inhibitory effect on Bax protein expression may be attenuated by Kaiso interaction with other proteins like p120^ctn^. Kaiso may also activate c-Myc, Cyclin D1 and BRCA1 expression independently or in collaboration with other cofactors in TNBC cells, which would also promote TNBC cell growth and survival

Conversely, in non-TNBC cells that express wt-p53, Kaiso exhibits a pro-apoptotic role (Supplementary Figure 2B), which is consistent with reports in other cell types that demonstrated a pro-apoptotic role for Kaiso in a wt-p53-dependent manner.^31^ Based on these findings, we surmise that the distinct roles of Kaiso in apoptosis may be due to its ability to interact with both wt-p53 and mut-p53 as shown in Figure 4a and Supplementary Figure 3A (see model indicated in Figure 8a). Indeed, several recent studies have reported differential activities of transcription factors that interact with both wt-p53 and mut-p53, reviewed in Kim *et al.*^53^ As Kaiso behaved in an anti-apoptotic manner in TNBC cells lacking wt-p53, we postulate that Kaiso may only function in a pro-apoptotic manner in BCa cells expressing wt-p53.

An unexpected finding of this study was that Kaiso depletion reduced BRCA1 expression at both the transcript and protein levels in TNBC cells, suggesting that BRCA1 may be a Kaiso target gene. Indeed, we observed that Kaiso associates with the BRCA1 promoter in both MDA-231 and Hs578T cells (Figure 6d) but more importantly, we also found that high Kaiso and BRCA1 expression correlates with poor overall survival in TNBC patients, as well as all BCa cases in general (Figures 7a and b). Collectively, our findings suggest that Kaiso may augment the survival and aggressiveness of TNBC cells by promoting BRCA1 expression (see model, Figure 8b). Hence, our demonstration that Kaiso-depletion enhanced the sensitivity of TNBC cells to the chemotherapy drug Cisplatin raise the exciting possibility that Kaiso may be a target for TN tumors with BRCA1 expression.

Together, this study reveals an essential role for Kaiso in the growth and survival of TNBC cells and suggests that Kaiso could be targeted for the treatment of a subset of triple negative tumors especially those expressing BRCA1. Future experiments (e.g. ChIP-sequencing and RNA-sequencing of control and Kaiso-depleted TNBC cells) are needed to fully delineate and understand the molecular mechanisms and signaling pathways that Kaiso participates in to contribute to the pathogenesis and survival of triple negative tumors.

## Materials and methods

### Cell culture

The human triple negative breast tumor cell lines MDA-MB-231 (hereafter MDA-231) and Hs578T, and their stable Kaiso-depleted (sh-K1 and sh-K2) derivatives were cultured as previously described.^26^ The non-TNBC cell line MCF-7 was purchased from ATCC (Manassas, VA, USA), while the triple negative breast tumor cell line MDA-MB-157 (hereafter MDA-157) and the non-TNBC cell line ZR75.1 were a kind gift from Dr. John Hassell (McMaster University, Hamilton, Canada). These cells were cultured as previously described.^54^ All cells were grown in 5% CO_2_ at 37 °C.

### Generation of stable Kaiso-depleted MDA-157 cell lines

Depletion of Kaiso in MDA-157 cells was achieved by stably transfecting cells with a pRetroSuper (pRS) vector containing shRNAs that targeted the Kaiso mRNA sequences; 5′-AAAAGATCATTGTTACCGATT-3′ and 5′-TTTTAACATTCATTCTTGGGAGAAG-3′ termed sh-K1 and sh-K2 as previously described.^26^ Stable control (transfected with a pRS-Kaiso scrambled shRNA)^26^ and Kaiso-depleted MDA-157 cells were maintained in media containing 1.0 *μ*g/ml of Puromycin (Invitrogen, Carlsbad, CA, USA). Only the most efficient Kaiso-depleted cells were selected for further analysis (Supplementary Figure 1b).

### Cell proliferation assay

Equal numbers (1 × 10^4^) of control and Kaiso-depleted (sh-K1 and sh-K2) MDA-231 and Hs578T cells were grown in 24-well plates for 3 days. Direct cell counts were obtained each day using the BioRAD TC10 automated cell counter and averaged using Microsoft Excel. Graphical representation of counts was achieved using GraphPad Prism software (La Jolla, CA, USA).

### MTT assay

Equal numbers (1 × 10^4^) of control and Kaiso-depleted (sh-K1 and sh-K2) MDA-231 and Hs578T cells were grown in duplicate in 96-well plates for 22 h. Cells were then immediately treated with MTT (3-(4,5-di**m**ethyl**t**hiazol-2-yl)-2, 5-diphenyl-**t**etrazolium bromide; Sigma Aldrich, USA) for 2 h. The precipitated formazan crystals were subsequently dissolved with 100 *μ*l of dimethyl sulfoxide and the optical density of the resulting reaction solution measured at 570 nm using the SpectraMax Plus 384 Microplate reader (Molecular Devices, Sunnyvale, CA, USA).

### Colony formation and soft agar assay

5 × 10^2^ control and Kaiso-depleted MDA-231 and Hs578T cells were cultured in 60 mm dishes in duplicate and allowed to grow and form colonies for 10–14 days. For the soft agar assays, 5 × 10^4^ control and Kaiso-depleted MDA-231 and Hs578T cells were cultured in 0.3% Agarose in 60 mm dishes, and allowed to grow and form colonies for 10 days. After the incubation period, colonies were stained with 0.5 and 0.05% Gentian Violet diluted in methanol for the colony formation and soft agar assays respectively. Images of colonies from the colony formation assay were obtained by using a Canon digital camera and then colonies were counted manually. For the soft agar assay, 10 × images of colonies were obtained using the Zeiss Axiovert 200 microscope (Carl Zeiss Canada Ltd., ON, Canada), and then counted using the ImageJ software. Graphical representation of counts (colony numbers) was achieved using GraphPad Prism software.

### Xenograft studies

All mice studies were approved by the Animal Research Ethics Board, McMaster University (AUP# 14–05–14) and performed in accordance with the guidelines of the Animal Research Ethics Board as previously described.^26^ In brief, equal numbers (4.5 × 10^6^) of control and Kaiso-depleted MDA-231 cells were injected subcutaneously into the mammary fat pad of ~5–8-week-old female NOD SCID gamma mice (*n*=5 each) and allowed to form tumors. Tumor growth was monitored using vernier calipers and tumor volume measurements calculated as previously described.^26^ Mice were euthanized at endpoint (tumor size 3300 mm^3^) as previously described^26^ and tumor tissues harvested for histological examination and IHC analyses.

### Immunohistochemistry

5 *μ*M sections of harvested MDA-231 xenografted tumor tissues were stained with mouse monoclonal antibody against Ki-67 (BD Biosciences; 1:50), rabbit monoclonal antibody against PCNA (**C**ell Signaling **T**echnology (CST)-D3H8P; 1:30 000), rabbit monoclonal antibody against c-Myc (Abcam; 1:100), rabbit monoclonal antibody against Cyclin D1 (CST-2978; 1:100) or rabbit monoclonal antibody against cleaved Caspase 3 (CST-9661; 1:50) overnight at 4 °C as previously described.^26^ Images were captured using the Aperio Slide scanner (Leica Biosystems, ON, Canada). Ki-67, PCNA and cleaved Caspase 3 counts were obtained from 5 different fields that represented staining observed in whole-tissue sections. The stained cells in these fields were counted blindly and independently by 2 students. Bar graphs representing counts were generated using GraphPad Prism software. Statistically analyses were also conducted using GraphPad Prism statistical software.

### Transient transfection assay and rescue experiments

MCF-7 and ZR75.1 parental cells were transfected with either a pCDNA3-empty vector (empty), or a pCDNA3 vector containing the sequence that encodes a sh-resistant mKaiso cDNA using the Turbofect transfection reagent (Thermo Scientific, Waltham, MA, USA) according to the manufacturer's instructions. 48 or 72 h post transfection, cells were either subjected to IB analysis, or treated with Geneticin (Invitrogen) at 250 *μ*g/ml for MCF-7 cells and 750 *μ*g/ml for ZR75.1 cells to select for efficient Kaiso overexpression. Three to four weeks post transfection, whole-cell lysates were obtained from the pCDNA3-empty and mKaiso transfected cells and subjected to IB analysis of interested proteins.

For rescue of Kaiso overexpression, pCDNA3 vector expressing the mKaiso cDNA coding sequence that is not targeted by the Kaiso-specific shRNA was transfected into MDA-231 and Hs578T sh-K2 (or sh-K) cells using the Turbofect transfection reagent (Thermo Scientific) as per the manufacturer's protocol. 24 or 48 h post transfection, cells were treated with Puromycin (Invitrogen) at 0.8 *μ*g/ml and Geneticin (Invitrogen) at 1000 *μ*g/ml for MDA-231 sh-K cells and Puromycin (Invitrogen) at 1.5 *μ*g/ml and Geneticin (Invitrogen) at 1000 *μ*g/ml for Hs578T sh-K cells to select for efficient Kaiso overexpression. Three to four weeks post transfection, MDA-231 and Hs578T sh-K (empty and mKaiso) cells were subjected to Annexin V-FITC staining. Whole-cell lysates were also obtained from MDA-231 and Hs578T sh-K (empty and mKaiso) cells and subjected to IB analysis of interested proteins.

### Caspase 3 assay

The Caspase 3 assay (colorimetric) kit was purchased from Abcam (Boston, MA, USA), and the assay performed according to the manufacturer's instructions. In brief, 1 × 10^6^ control and Kaiso-depleted (sh-K1 and sh-K2) MDA-231 and Hs578T cells were re-suspended in 50 *μ*l of chilled cell lysis buffer, incubated on ice for 10 min and pelleted by centrifugation at 13 000 r.p.m. for 1 min. The resulting supernatant (cytosolic extract) was then transferred to a new tube, quantified and then~200 *μ*g of protein per 50 *μ*l cell lysis buffer transferred into 96-well plates in duplicate per cell condition. 50 *μ*l cell lysis buffer without protein samples was also aliquoted into 96-well plates to provide background readings. 50 *μ*l reaction buffer (2 ×) containing 10 mM DTT was added to each well containing experimental samples (in duplicate) and cell lysis buffer (without samples) followed by 5 *μ*l of 4 mM DEVD-p-NA substrate (200 *μ*M final concentration). The resultant mixture was incubated at 37 °C for 2 h, and then the optical density of the solution was measured at 405 nm using the SpectraMax Plus 384 Microplate reader (Molecular Devices).

### ANNEXIN V-FITC staining assay

The FITC–conjugated Annexin V apoptosis detection kit was purchased from Abcam, and staining performed according to the manufacturer's instructions. In brief, equal numbers (1 x10^5^) of Ctrl and sh-K MDA-231, Hs578T and MDA-157 cells, MDA-231 and Hs578T sh-K (empty and mKaiso) cells, as well as MCF-7 (empty and mKaiso) cells were re-suspended in 1 × binding buffer (Abcam) after being washed with 1 × PBS and trypsinized with 1 × Trypsin (Invitrogen). These cells were subsequently stained with Annexin V-FITC and propidium iodide (PI) and incubated for 10 min prior to analysis by Flow cytometry. Data were acquired using the LSRFortessa flow cytometer (BD Biosciences, Mississauga, Canada) and analyzed with FlowJo version 9 software.

### Quantitative reverse transcription-PCR (qRT-PCR)

qRT-PCR experiments were conducted as previously described^26^ using the following primers: Bax forward: 5′-GCCCTTTTGCTTCAGGGTTT-3′ and reverse: 5′-GCAATCATCCTCTGCAGCTC-3′ at 60 °C, PUMA forward: 5′-AGCAGGGCAGGAAGTAACAA-3′ and reverse: 5′-CCCTGGGGCCACAAATCT-3′ at 55 °C, BRCA1 forward: 5′-CTCGCTGAGACTTCCTGGAC-3′ and reverse: 5′-TCAACTCCAGACAGATGGGAC-3′ at 62 °C. The SensiFAST cDNA synthesis kit and the SensiFAST SYBR Hi-ROX kit (FroggaBio Scientific Solutions, Toronto, ON, Canada) were used in place of the qScript cDNA SuperMix and Perfecta SYBR Green SuperMix ROX (Quanta BioSciences, Gaithersburg, MD, USA) as previously described.^26^

### ChIP and ChIP-PCR

ChIP and ChIP-PCR were performed as previously described.^26^ The following primers were used to amplify a minimal Bax, PUMA and BRCA1 promoter region, respectively, containing one or more KBS: Bax KBS forward: 5′-CTAATTCCTTCTGCGCTGGG-3′, and reverse: 5′-GTCCAATCGCAGCTCTAATGC-3′ at 64 °C; PUMA KBS forward: 5′-GATCGAGACCATCCTGGCTA-3′ and reverse: 5′-CGATCTCAGCAAACTGCAAG-3′ at 64 °C; and BRCA1 KBS forward: 5′-AGGGCTCTCTCATCCTGTCA-3′ and reverse: 5′-TGTCCGCCATGTTAGATTCA-3′ at 64 °C.

### Immunoprecipitation

Whole-cell lysates were immunoprecipitated with anti-Kaiso 6F mouse monoclonal antibody,^55^ anti-p53 mouse monoclonal antibody (CST-2524, which recognizes both wt and mutant-p53), anti-p53 rabbit monoclonal antibody (Abcam-ab32049 that recognizes only mutant-p53) or normal rabbit IgG antibody (Santa Cruz Technology) for 2 h or overnight at 4 °C. The immuno-precipitates were collected by incubation with 50 *μ*l Protein A agarose beads that were subsequently washed five times with lysis buffer before proceeding to SDS-PAGE and IB analysis.

### Immunoblot and densitometry analysis

IB analysis was performed as previously described.^26^ Overnight incubations were performed at 4 °C using the following primary antibodies at their respective dilutions; Kaiso-specific rabbit polyclonal (gift from Dr. Reynolds; 1:5000), mouse monoclonal antibody against c-Myc (SantaCruz (9E10); 1:500), rabbit polyclonal antibody against Cyclin D1 (US Biological (144418); 1:5000), p120^ctn^-15D2 specific mouse monoclonal (gift from Dr. Reynolds; 1:1000^56^), Bax-specific rabbit monoclonal (1:500; CST-5023), PUMA-specific rabbit monoclonal (1:500; CST-12450), p53-specific rabbit polyclonal (1:2000; Abcam-ab32049), cleaved PARP-specific rabbit monoclonal (1:1000; CST-5625), BRCA1-specific rabbit polyclonal (1:2000; Abcam-ab131360) and mouse anti-*β*-actin monoclonal (1:50 000; Sigma Aldrich). IB images were obtained using the Bio-Rad ChemiDoc MP imaging system (Bio-Rad Laboratories, Mississauga, ON, Canada). The optical densities of Kaiso, c-Myc, Cyclin D1, p120-1, p120-3, p53, Bax, PUMA and *β*-actin signals were quantified and analyzed using the Image Lab software (Bio-Rad), while the relative ratio of Kaiso/*β*-actin, c-Myc/*β*-actin, Cyclin D1/*β*-actin, p120-1/*β*-actin, p120-3/*β*-actin, p53/*β*-actin, Bax/*β*-actin and PUMA/*β*-actin were calculated as indicated using Microsoft Excel. Graphical representation of each respective value was accomplished using GraphPad Prism software.

### Gene expression analysis of GEO data sets

Gene expression analyses were conducted on five publicly available data sets obtained using Affymetrix HG-U133 plus 2.0 gene chip arrays (Affymetrix, Santa Clara, CA, USA). The transcript profiles of these data sets were deposited in the GEO database under accession numbers GSE20685, GSE21653, GSE16446, GSE19615 and GSE9195.^57, 58, 59, 60, 61, 62^ All samples used for this study were normalized with frozen robust multi-array analysis^63^ and then the distance-weighted discrimination method^64^ was used to remove technical variation from the data sets that were to be combined. The combined data sets correlation coefficients for pair-wise comparisons of samples using Affymetrix house-keeping probe sets were computed, and only samples exhibiting a correlation higher than 0.95 with at least half of the data set were selected for further classification. This resulted in a cohort containing 894 tumor samples, which was subsequently used for generating Kaplan–Meier survival curves and performing log-rank analysis.

### Gene expression analysis of TCGA data sets

TCGA Level 3 IlluminaHiSeq_RNASeqV2 expression (Illumina, Inc., San Diego, CA, USA) and associated clinical data were downloaded for all available patients from the Broad GDAC Firehose repository (https://gdac.broadinstitute.org/) on 16 September 2016 (*n*=1212). In all further analyses this data set is referred to as ‘TCGA dataset'. We selected tumor samples only (*n*=1,094), and their RSEM-quantified gene expression values were log2-transformed and used for further analyses to represent gene expression. For identification of ER, PR and ERBB2 status and for overall survival information we used the downloaded clinical data. All data processing was performed using R software.^65^

### Survival analysis

Survival analysis and visualization of the Kaplan–Meier curves were performed using GraphPad Prism statistical software (GraphPad Software, Inc., La Jolla, CA, USA). For statistical tests *P*-value<0.05 indicated significance.

### Statistical analyses

All statistical analyses were performed as previously described^26^ using the GraphPad Prism software (GraphPad Software, Inc., La Jolla, CA, USA). *P*<0.05 values were considered statistically significant and data are presented as means±S.E.M.
