# Supplementary material for: Kaiso depletion attenuates the growth and survival of triple negative breast cancer cells
Source: Cell Death Dis. 2017 Mar 23;8(3):e2689–. doi: 10.1038/cddis.2017.92 (PMC5386582; doi:10.1038/cddis.2017.92)
Supplement: Supplementary Figure 1 [file cddis201792x1.pdf]

A.

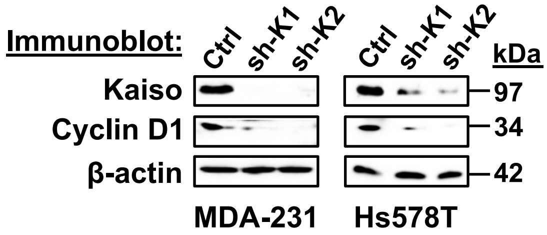

B.

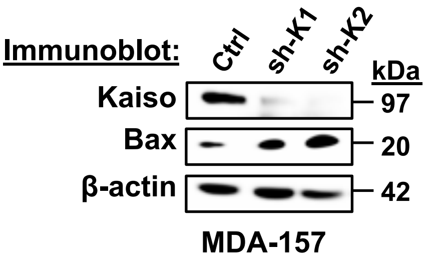

Supp. Figure 1: ***Characterization of Kaiso-depleted TNBC cells.*** **(A)** Immunoblot analysis of MDA-231 and Hs578T cells show the efficient generation of Kaiso-depleted MDA-231 and Hs578T (sh-K1 and sh-K2) cells. The most efficient clones (sh-K1 and sh-K2) were selected for further analysis, and immunoblot analysis of these clones shows decreased expression of Cyclin D1 in MDA-231 and Hs578T cells. **(B)** Immunoblot analysis of MDA-157 cells shows the efficient generation of Kaiso-depleted MDA-157 (sh-K1 and sh-K2) clones which exhibited increased expression of Bax. Data shown is representative of three independent experiments.
