# Supplementary material for: Kaiso depletion attenuates the growth and survival of triple negative breast cancer cells
Source: Cell Death Dis. 2017 Mar 23;8(3):e2689–. doi: 10.1038/cddis.2017.92 (PMC5386582; doi:10.1038/cddis.2017.92)
Supplement: Supplementary Figure 2 [file cddis201792x2.pdf]

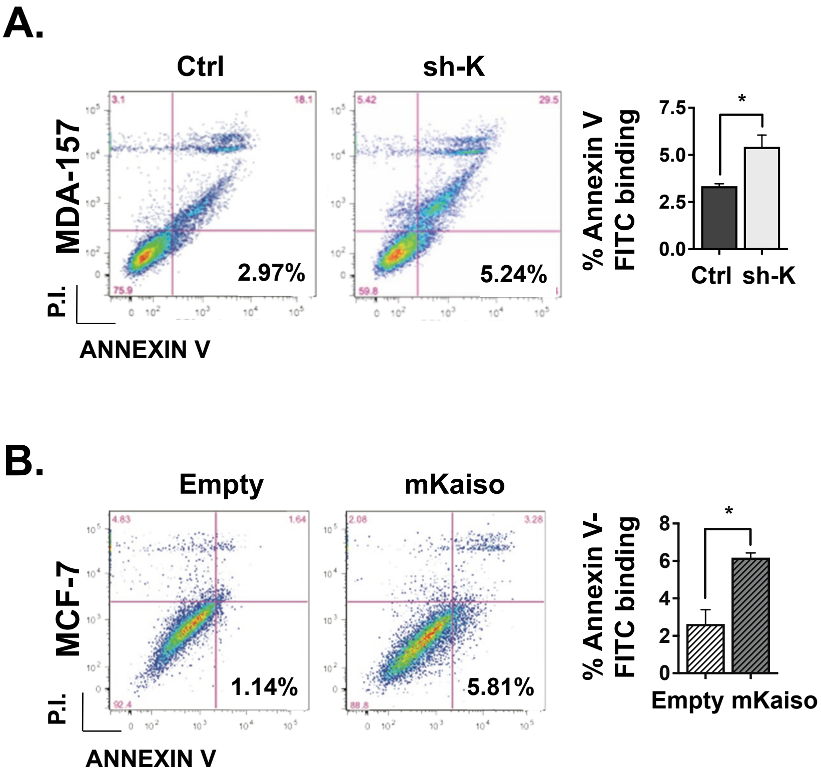

Supp. Figure 2: ***Kaiso roles in apoptosis is influenced by the status of p53 expressed in the respective cells.*** **(A)** Kaiso-depleted (sh-K) MDA-157 cells exhibit increased apoptosis compared to control (Ctrl) cells as detected by Annexin V-FITC staining. **(B)** Kaiso overexpression in the non-TNBC breast cancer cell line MCF-7 results in increased apoptosis as measured by Annexin V-FITC staining. Data shown is representative of at least three independent experiments.
