# Supplementary material for: Kaiso depletion attenuates the growth and survival of triple negative breast cancer cells
Source: Cell Death Dis. 2017 Mar 23;8(3):e2689–. doi: 10.1038/cddis.2017.92 (PMC5386582; doi:10.1038/cddis.2017.92)
Supplement: Supplementary Figure 3 [file cddis201792x3.pdf]

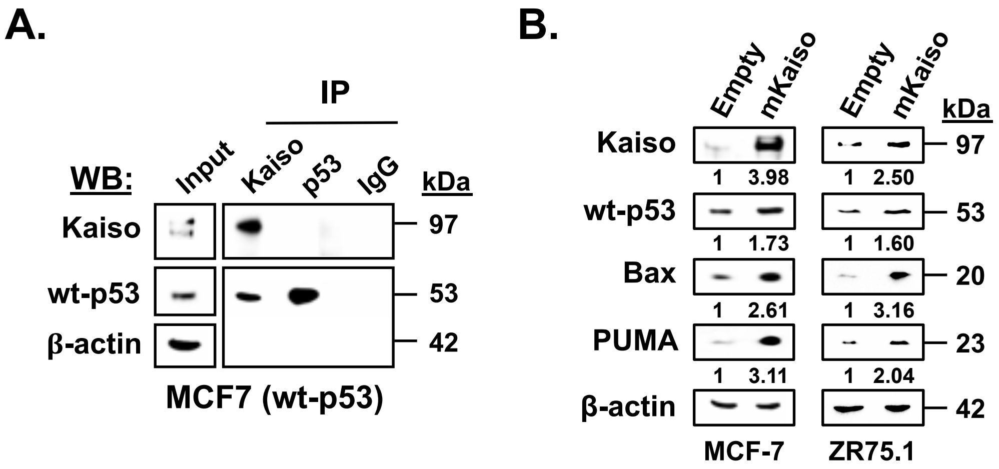

Supp. Figure 3: ***Kaiso interacts with wild-type p53 and promotes expression of pro-apoptotic Bax and PUMA proteins and cell death in wild-type p53-expressing breast cancer cells.*** **(A)** Immunoprecipitation and immunoblot analysis revealed that Kaiso interacts with **w**ild-**t**ype (wt) p53 in MCF-7 cells. **(B)** Kaiso overexpression (mKaiso) in the non-TNBC wt-p53 expressing breast cell lines MCF-7 and ZR75.1 results in increased Bax and PUMA protein expression as detected by immunoblot analysis. Numbers below each blot represent fold-change in expression. Data shown is representative of at least three independent experiments.
