# Supplementary material for: Kaiso depletion attenuates the growth and survival of triple negative breast cancer cells
Source: Cell Death Dis. 2017 Mar 23;8(3):e2689–. doi: 10.1038/cddis.2017.92 (PMC5386582; doi:10.1038/cddis.2017.92)
Supplement: Supplementary Figure 4 [file cddis201792x4.pdf]

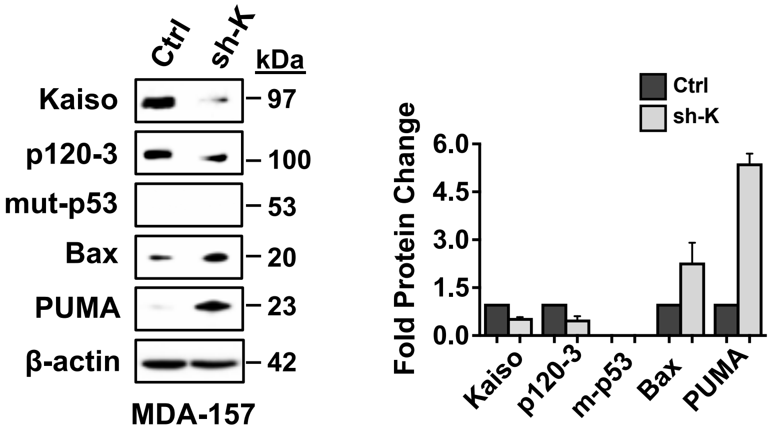

Supp. Figure 4: ***Kaiso-depletion results in increased expression of pro-apoptotic proteins in MDA-157 cells.*** Immunoblot images and densitometry analysis show that Kaiso-depleted MDA-157 cells express increased amounts of Bax and PUMA protein compared to control cells. Data shown is representative of at least three independent experiments.
