# Supplementary material for: Kaiso depletion attenuates the growth and survival of triple negative breast cancer cells
Source: Cell Death Dis. 2017 Mar 23;8(3):e2689–. doi: 10.1038/cddis.2017.92 (PMC5386582; doi:10.1038/cddis.2017.92)
Supplement: Supplementary Figure 5 [file cddis201792x5.pdf]

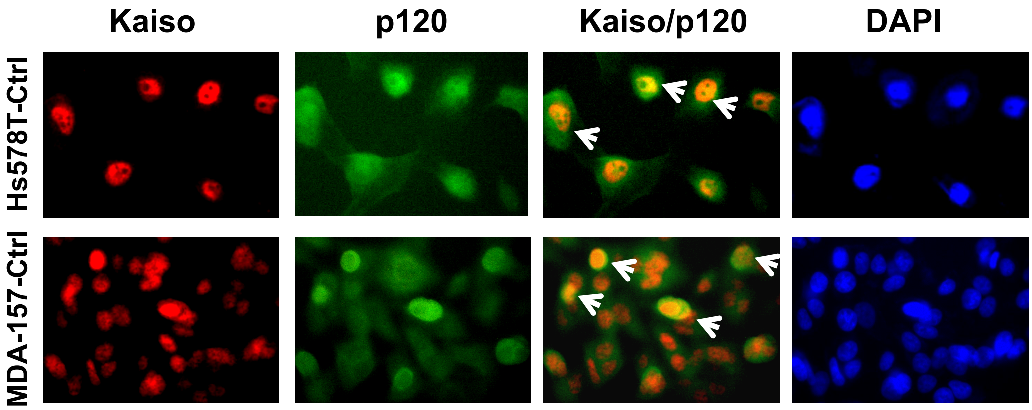

Supp. Figure 5: *Kaiso and p120^{ctn} co-localizes in Hs578T and MDA-157 cells.* (A) Immunofluorescence images show the co-localization of Kaiso and p120^{ctn} in some but not all parental Hs578T and MDA-157 cells as indicated by the white arrowheads. Kaiso and p120 appear to co-localize more in Hs578T cells compared to MDA-157 cells. Data shown is representative of three independent experiments.
