# Supplementary material for: Kaiso depletion attenuates the growth and survival of triple negative breast cancer cells
Source: Cell Death Dis. 2017 Mar 23;8(3):e2689–. doi: 10.1038/cddis.2017.92 (PMC5386582; doi:10.1038/cddis.2017.92)
Supplement: Supplementary Figure 6 [file cddis201792x6.pdf]

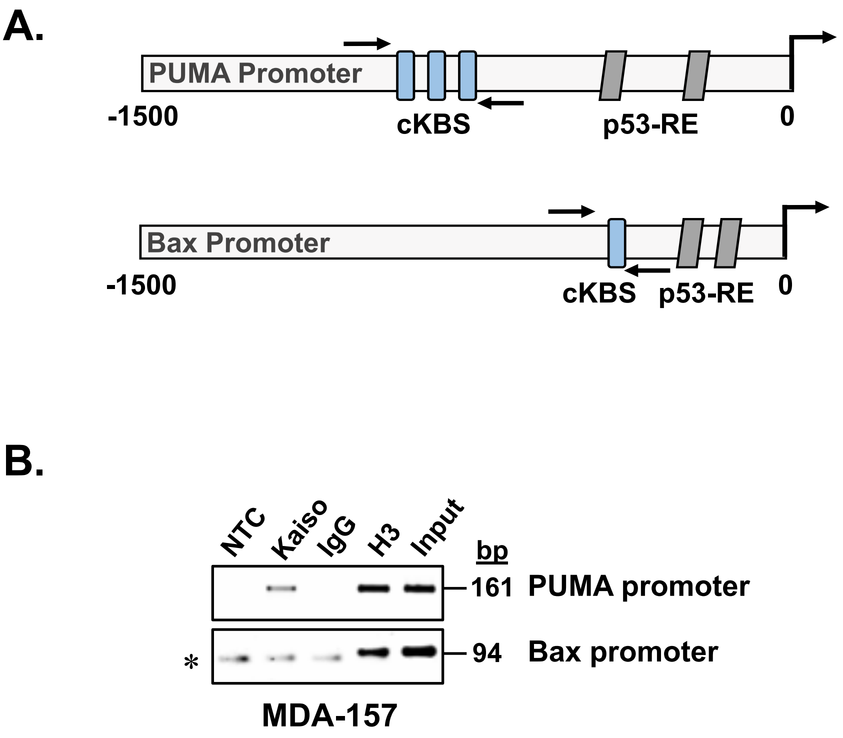

Supp. Figure 6: ***Kaiso associates endogenously with the PUMA promoter in MDA-157 cells.*** **(A)** Schematic illustration of the Bax and PUMA promoters showing the presence of several **c**ore **K**aiso **b**inding **s**equences (**cKBS**). **(B)** ChIP-PCR analysis of MDA-157 chromatin revealed that Kaiso interacts with the PUMA promoter but not the Bax promoter. Data shown are representative of three independent experiments. The asterisk (*) indicates primer dimers in the Bax promoter ChIP.
